# Neutrophil-activating Peptide 2 as a Novel Modulator of Fibrin Clot Properties in Patients with Atrial Fibrillation

**DOI:** 10.1007/s12975-023-01165-1

**Published:** 2023-06-09

**Authors:** Michał Ząbczyk, Joanna Natorska, Paweł T. Matusik, Patrycja Mołek, Wiktoria Wojciechowska, Marek Rajzer, Renata Rajtar-Salwa, Tomasz Tokarek, Aleksandra Lenart-Migdalska, Maria Olszowska, Anetta Undas

**Affiliations:** 1 Department of Thromboembolic Disorders, Institute of Cardiology, Jagiellonian University Medical College, Pradnicka 80, 31-202 Krakow, Poland; 2 Krakow Centre for Medical Research and Technologies, the John Paul II Hospital, Pradnicka 80, Krakow, Poland; 3 Institute of Cardiology, Faculty of Medicine, Jagiellonian University Medical College, Pradnicka 80 Kraków, Poland; 4 Department of Electrocardiology, the John Paul II Hospital, Pradnicka 80, Kraków, Poland; 5 1st Department of Cardiology, Interventional Electrocardiology and Arterial Hypertension, Jagiellonian University Medical College, Jakubowskiego 2, Kraków, Poland; 6 Department of Cardiology and Cardiovascular Interventions, University Hospital, Jakubowskiego 2, Krakow, Poland; 7 Center for Invasive Cardiology, Electrotherapy and Angiology, Kilinskiego 68, Nowy Sacz, Poland; 8 Center for Innovative Medical Education, Jagiellonian University Medical College, Medyczna 9, Krakow, Poland; 9 Department of Cardiac and Vascular Diseases, Faculty of Medicine, Institute of Cardiology, Jagiellonian University Medical College, John Paul II Hospital, Pradnicka 80, Kraków, Poland

**Keywords:** Atrial fibrillation, Fibrin clot, Neutrophil-activating peptide 2, Oxidative stress

## Abstract

Neutrophil-activating peptide 2 (NAP-2, CXCL7), a platelet-derived neutrophil chemoattractant, is involved in inflammation. We investigated associations between NAP-2 levels, neutrophil extracellular traps (NETs) formation, and fibrin clot properties in atrial fibrillation (AF). We recruited 237 consecutive patients with AF (mean age, 68 ± 11 years; median CHA_2_DS_2_VASc score of 3 [2–4]) and 30 apparently healthy controls. Plasma NAP-2 concentrations were measured, along with plasma fibrin clot permeability (K_s_) and clot lysis time (CLT), thrombin generation, citrullinated histone H3 (citH3), as a marker of NETs formation, and 3-nitrotyrosine reflecting oxidative stress. NAP-2 levels were 89% higher in AF patients than in controls (626 [448–796] vs. 331 [226–430] ng/ml; p < 0.0001). NAP-2 levels were not associated with demographics, CHA_2_DS_2_-VASc score, or the AF manifestation. Patients with NAP-2 in the top quartile (> 796 ng/ml) were characterized by higher neutrophil count (+ 31.7%), fibrinogen (+ 20.8%), citH3 (+ 86%), and 3-nitrotyrosine (+ 111%) levels, along with 20.2% reduced K_s_ and 8.4% prolonged CLT as compared to the remaining subjects (all p < 0.05). NAP-2 levels were positively associated with fibrinogen in AF patients (r = 0.41, p = 0.0006) and controls (r = 0.65, p < 0.01), along with citH3 (r = 0.36, p < 0.0001) and 3-nitrotyrosine (r = 0.51, p < 0.0001) in the former group. After adjustment for fibrinogen, higher citH3 (per 1 ng/ml β = -0.046, 95% CI -0.029; -0.064) and NAP-2 (per 100 ng/ml β = -0.21, 95% CI -0.14; -0.28) levels were independently associated with reduced K_s_. Elevated NAP-2, associated with increased oxidative stress, has been identified as a novel modulator of prothrombotic plasma fibrin clot properties in patients with AF.

## Introduction

Atrial fibrillation (AF) is the most common sustained cardiac arrhythmia with increasing incidence with aging [1, 2]. AF is associated with a prothrombotic state, enhanced oxidative stress, and neutrophil extracellular traps (NETs) formation [3, 4]. In AF, all these factors have been linked with unfavourably altered fibrin clot structure and function, typically evidenced by reduced clot permeability (K_s_) and susceptibility to fibrinolysis measured in vitro [4, 5]. The prothrombotic clot phenotype involves formation of more compact fibrin networks composed of thinner and more branched fibrin fibres, which are more resistant to tissue plasminogen activator-mediated lysis. Fibrin clot phenotype is modulated by multiple genetically determined and acquired factors, including fibrinogen [6]. Posttranslational modifications of fibrinogen, including oxidation, carbonylation or nitration have been identified as mechanisms altering fibrin clot properties [7]. Oxidative stress is also linked with neutrophil activation. The redox imbalance of neutrophils during the oxidative burst promotes NETs generation [8]. Of note, increased NETs markers have been shown to be associated with prothrombotic fibrin clot properties and identified AF patients at high thromboembolic risk [4]. Given data indicating that prothrombotic clot properties can predict stroke and bleeding risk in AF patients despite oral anticoagulation [9–11], complex mechanisms leading to unfavorable clot characteristics in anticoagulated AF patients need to be explored.

Neutrophil-activating peptide 2 (NAP-2), also called chemokine Cys-X-Cys motif ligand 7 (CXCL7) is a primary neutrophil chemoattractant [12]. Large amounts of its precursor, connective tissue-activating peptide III (CTAP-III) are stored in platelet alpha granules. Upon platelet activation CTAP-III is proteolysed by neutrophilic cathepsin G and released as NAP-2. NAP-2 induces neutrophil migration and activation [12]. In a mouse model, NAP-2 chemotactic gradient that guides neutrophils to the site of vascular injury has been demonstrated within the thrombus body [13, 14]. Pharmacologic blockade of CXC chemokine receptor 1/2 (CXCR1/2) and NAP-2 genetic deficiency resulted in defective leukocyte intrathrombus migration at sites of vessel injury [13]. Kollikowski et al. [15] showed in patients with acute ischemic stroke a significant role of NAP-2 in cerebral ischemia, neutrophil activation and its association with myeloperoxidase concentration, released by neutrophils, which is also associated with NETosis. Stable angina patients had markedly higher plasma levels of NAP-2 and increased expression of the monocyte CXCR2 compared to healthy controls [16]. Elevated NAP-2 levels have also been reported in patients with critical limb ischemia [17], antiphospholipid syndrome (APS) [18], and colorectal cancer [19, 20]. Increased expression of genes related to platelet activation, including *PPBP/CXCL7*, has been shown in coronavirus disease 2019 (COVID-19) patients [21].

To the best of our knowledge, NAP-2 has not been evaluated in AF patients and its potential impact on a prothrombotic state in this disease is unknown. The aim of the study was to establish whether NAP-2 present in circulating blood can contribute to thrombin generation and the prothrombotic clot phenotype in AF patients.

## Methods

### Patients

A total of 237 consecutive adult patients with documented AF, who were referred to the John Paul II Hospital and the University Hospital, Krakow, Poland, between June 2020 and December 2021 were recruited. Exclusion criteria were as follows: acute myocardial infarction (MI), stroke or venous thromboembolism within the preceding 3 months, heart failure in New York Heart Association (NYHA) class III or IV, stage 5 chronic kidney disease, liver injury, known cancer, pregnancy, autoimmune diseases, signs of acute infection, C-reactive protein (CRP) > 10 mg/l, and use of vitamin K antagonists. Follow-up data of patients was collected at least twice a year by telephone or through clinic visits. The primary endpoint was the documented occurrence of thromboembolic events based on the clinical symptoms. Major bleeding was the secondary endpoint.

The CHA_2_DS_2_-VASc score was used to assess the risk of stroke in AF patients [22]. HAS-BLED score was used to evaluate the risk of major bleeding [22]. AF classification based on clinical presentation, duration, and spontaneous termination was documented at enrolment according to the 2020 European Society of Cardiology (ESC) Guidelines [22]. Hypercholesterolemia was diagnosed based on medical records, cholesterol-lowering therapy, or low-density lipoprotein cholesterol levels > 3.0 mmol/l [23]. Arterial hypertension was diagnosed based on a history of hypertension (blood pressure ≥ 140/90 mmHg) or preadmission antihypertensive treatment. Type 2 diabetes was diagnosed based on fasting serum glucose ≥ 7.0 mmol/l on two separate occasions, HbA_1c_ ≥ 48 mmol/mol (6.5%), or post-load plasma glucose levels ≥ 11.1 mmol/l. Prior ischemic stroke was diagnosed based on the symptoms and positive findings of computed tomography or magnetic resonance imaging (MRI), based on World Health Organization (WHO) criteria. Thirty healthy individuals of similar age, sex, and body mass index (BMI) served as controls. The study was approved by the Ethics Committee of Jagiellonian University and performed in accordance with the relevant guidelines and regulations. All study participants provided written informed consent prior to their enrolment.

### Laboratory Investigations

Fasting venous blood was drawn during anticoagulant therapy with direct oral anticoagulants (DOAC) and time since the last dose of DOAC was noted. Plasma samples from patients, in whom plasma DOAC levels were > 30 ng/ml, were treated with the DOAC-Stop (Haematex Research, Sydney, Australia) prior to coagulation assessment to eliminate potential residual anticoagulant effects. Blood was drawn from the antecubital vein into citrated tubes and centrifuged at 2500 g at 20 °C for 20 min or into serum tubes and centrifuged at 1600 g at 4 °C for 10 min. Aliquots were stored at -80 °C. Blood cell count, creatinine, fibrinogen, lipid profile, glucose, and CRP were assayed by routine laboratory techniques. ELISA kits were used to quantify citrullinated histone H3 (citH3), as a circulating stable NETosis marker (Cayman Chemical, Ann Arbor, MI, USA), plasminogen activator inhibitor-1 (PAI-1) antigen, thrombin activatable fibrinolysis inhibitor (TAFI) (both Hyphen-Biomed, Neuville-Sur-Oise, France), soluble P-selectin (R&D, Minneapolis, MN, USA), plasma 3-nitrotyrosine, a marker of oxidative stress (OxiSelect, Cell Biolabs Inc., San Diego, USA), and plasma NAP-2 (Invitrogen, Thermo Fisher, Waltham, MA, USA).

### Endogenous Thrombin Potential

Calibrated automated thrombogram (CAT; Thrombinoscope BV, Maastricht, Netherlands) was performed according to the manufacturer’s instructions in the 96-well plate fluorometer (Ascent Reader, Thermolabsystems OY, Helsinki, Finland), equipped with the 390/460 filter set, at a temperature of 37 °C. Briefly, to 80 µL platelet-poor plasma 20 µL of tissue factor (TF)-based activator (PPP Reagent; final TF concentration, 5 pM) and FluCa solution (both Diagnostica Stago) were added. Each plasma sample was analyzed in duplicate. The maximum concentration of thrombin formed during the recording time is described as the peak thrombin generated and the area under the curve represents endogenous thrombin potential (ETP) [24]. The intra-assay variability was 8%.

### Fibrin Clot Analysis

Fibrin clot permeability was determined as described previously [4]. Briefly, CaCl_2_ (20 mM) and human thrombin (1 U/mL; Merck, Darmstadt, Germany) were mixed with citrated plasma. The permeation coefficient (K_s_) reflecting the average size of pores formed in the fibrin network. K_s_ was calculated as follows: K_s_ = Q × L × η/t × A × Δp. Q is the flow rate in percolating time (t), L is the length of a fibrin gel, η is the viscosity of liquid, A is the cross-sectional area, and Δp is a differential pressure. The interassay and intraassay coefficients of variation were < 7%. Fibrinolysis capacity was determined using CLT, as described previously [4]. Briefly, citrated plasma was mixed with 20 mM CaCl_2_, 0.5 U/ml thrombin (Merck), 15 µM phospholipid vesicles (Rossix, Mölndal, Sweden) and 18 ng/mL recombinant tissue plasminogen activator (Actilyse 20 mg, Boehringer Ingelheim, Germany). Absorbance was determined at 405 nm (Tecan Sunrise, Männedorf, Switzerland). We defined CLT as time from the midpoint of clear-to-maximum turbid transition, to the midpoint of maximum-turbid-to-clear transition. The intra-assay variability was 7%.

### Scanning Electron Microscopy (SEM) Analysis

Fibrin clots prepared as for the permeation analysis from plasma of randomly selected patients with similar fibrinogen levels and high (n = 10), median (n = 10), or low (n = 10) concentrations of NAP-2 were analyzed using SEM. Clots were fixed using 2.5% glutaraldehyde, washed, dehydrated, dried, and coated with gold. Samples were scanned in 10 areas (JEOL JCM-6000; JEOL Ltd., Tokyo, Japan) and a fibrin fiber diameter of at least 50 single fibers per clot was measured (ImageJ, US National Institutes of Health, Bethesda, MD, USA). Images were analyzed by two independent investigators unaware of the sample origin.

### Statistical Analysis

Continuous variables were expressed as mean ± standard deviation (SD) or median with interquartile range (IQR). Normality of the data was assessed using the Shapiro–Wilk test. Categorical variables were presented as numbers and percentages and were compared by two-sided Pearson’s χ^2^ or Fisher’s exact test. Differences between 2 groups were compared using the Student's t test for normally distributed continuous variables, while for non-normally distributed continuous variables the Mann–Whitney U test was used. Analysis of variance (ANOVA) was used to compare continuous variables between multiple groups. Associations between nonparametric or parametric variables were assessed by Spearman’s or Pearson’s tests, respectively. The univariable linear regression models were performed to identify associations between fibrin clot properties and demographic, clinical, and laboratory variables. Variables that were associated with K_s_ or CLT with a significance level of p-value < 0.2 in the univariable models or were clinically important were selected and the multivariable linear models were fitted using stepwise regression with minimization of the Bayesian information criterion (BIC) and adjusted for age, sex, and fibrinogen levels with K_s_ or CLT as dependent variables. Final models were validated using bootstrap resampling and by examination of the residuals. Variance Inflation Factors (VIF) were used to assess the multicollinearity. Univariate logistic regression models were performed to identify predictors of primary and secondary endpoints. A p-value of < 0.05 was considered statistically significant. Statistical analysis was performed using STATISTICA 13 (StatSoft STATISTICA, Poland 2022) and R 4.1.1 (The R Foundation for Statistical Computing, Vienna, Austria, 2021).

## Results

As shown in Table 1, we studied 104 (44%) patients with paroxysmal AF, 51 (21.5%) with persistent AF, and 72 (30.4%) with permanent AF (Table 1). As many as 74 (31.2%) patients were in sinus rhythm. Median time since the diagnosis of AF was 36 (IQR 24–84; range, 0.5–240) months. Median CHA_2_DS_2_VASc score was 3 (2–4), and 205 (86.5%) patients had at least 2 points in the score. Median HAS-BLED score was 1 (1–2). All patients were treated with DOACs, i.e. with rivaroxaban (41%), dabigatran (35.9%), and apixaban (23.1%) used at recommended doses. Median time from the last DOAC intake to blood draw was 9 (3–24) hours. AF patients compared to healthy controls had markedly increased citH3, 3-nitrotyrosine levels, thrombin generation capacity and were characterized by prothrombotic plasma fibrin clot phenotype (Table 1).

**Table 1:** Baseline characteristics of AF patients

Variable	Healthy controls N = 30	AF patients N = 237	NAP-2 > 796 ng/ml (top quartile), n = 57 (24%)	NAP-2 ≤ 796 ng/ml, n = 180 (76%)	*P*-value
Age, years	66 ± 9	68 ± 11	68 ± 13	69 ± 10	0.60
Male, n (%)	17 (56.7)	141 (59.6)	41 (71.9)	100 (55.6)	0.01
BMI, kg/m^2^	27 ± 4	29 ± 5	30 ± 4	29 ± 5	0.24
CHA_2_DS_2_VASc score	-	3 (2–4)	3 (1–4)	3 (2–4)	0.44
Paroxysmal AF, n (%)	-	104 (43.9)	22 (38.6)	82 (43.2)	0.54
Permanent AF, n (%)	-	72 (30.4)	21 (36.8)	51 (26.8)	0.14
Persistent AF, n (%)	-	40 (16.9)	12 (21.1)	28 (15.6)	0.33
Long-standing persistent AF, n (%)	-	11 (4.6)	2 (3.5)	9 (5)	0.64
AF duration, months	-	36 (24–84)	33 (24–84)	36 (18–84)	0.86
Active smoking, n (%)	2 (6.7)	14 (5.9)	3 (5.3)	11 (6.1)	0.55
Hypertension, n (%)	-	182 (76.8)	43 (75.4)	139 (73.2)	0.73
Hypercholesterolemia, n (%)	4 (13.3)	12 (5.1)	3 (5.3)	9 (5)	0.58
Diabetes, n (%)	-	56 (23.6)	18 (31.6)	38 (20)	0.067
Congestive heart failure, n (%)	-	37 (15.6)	10 (17.5)	27 (15)	0.64
Prior stroke, n (%)	-	23 (9.7)	4 (7)	19 (10)	0.50
Prior MI, n (%)	-	28 (11.8)	4 (7)	24 (12.6)	0.24
Previous VTE, n (%)	-	5 (2.1)	2 (3.5)	3 (1.7)	0.35
Vascular disease, n (%)	-	90 (38)	23 (40.4)	67 (37.2)	0.67
Medications, n (%)					
Rivaroxaban	-	98 (41.4)	24 (42.1)	74 (38.9)	0.67
Dabigatran	-	93 (35.2)	22 (38.6)	71 (39.4)	0.91
Apixaban	-	46 (19.4)	10 (17.5)	36 (20)	0.68
Statin	-	161 (67.9)	43 (75.4)	118 (62.1)	0.064
Aspirin	-	27 (11.4)	7 (12.3)	20 (11.1)	0.81
Clopidogrel	-	18 (7.6)	3 (5.3)	15 (8.3)	0.45
ACEI	-	158 (66.7)	31 (54.4)	127 (70.6)	0.024
Laboratory investigations					
WBC, 10^3^/µl	6.75 (5.87–7.97)	6.8 (5.92–7.92)	7.6 (6.37–9.05)	6.69 (5.73–7.42)	0.0012
Neutrophil count, 10^3^/µl	4.58 (3.49–6.05)	4.69 (3.89–5.49)	5.69 (3.96–6.63)	4.32 (3.89–5.23)	0.0026
Platelet count, 10^3^/µl	215 ± 89	219 ± 77	218 ± 72	219 ± 79	0.99
Hemoglobin, g/dl	13.9 ± 1.4	14.2 ± 1.3	14.2 ± 1.2	14.2 ± 1.3	0.99
Glucose, mM	5.5 ± 0.4	6.1 ± 1.7	6.1 ± 1.2	6.1 ± 1.8	0.83
Creatinine, µM	72 ± 16	91 ± 20*	88 ± 19	91 ± 21	0.42
eGFR, ml/min/1.72 m^2^	87 ± 19	70 ± 16*	73 ± 16	69 ± 16	0.15
C-reactive protein, mg/l	1.73 (0.94–3.87)	1.75 (1–4.38)	1.79 (1–4.83)	1.73 (1–4.44)	0.68
Fibrinogen, g/l	3.21 ± 1.07	3.34 ± 0.98	3.83 ± 1.16	3.17 ± 0.85	< 0.0001
P-selectin, ng/ml	32.2 (27.8–37.2)	36.5 (29.6–43.2)	32.2 (28.9–43.5)	37.6 (32.4–43.2)	0.30
citH3, ng/ml	0.9 (0.6–1.2)	4.1 (2.6–8.3)*	6.77 (3.57–11.3)	3.64 (2.16–6.99)	< 0.0001
NAP-2, µg/ml	331 (226–430)	626 (448–796)*	870 (825–897)	557 (399–683)	< 0.0001
ETP, nM x min	1247 (1089–1348)	2027 (1757–2209)*	1996 (1744–2207)	2043 (1758–2209)	0.89
Peak thrombin, nM	234 (228–276)	466 (402–507)*	463 (396–498)	466 (404–512)	0.80
TAFI, %	98 (87–110)	96 (85–109)	97.8 (84.9–116.8)	92.6 (85–106.8)	0.40
PAI-1 antigen, ng/ml	15.2 (12–24)	15.4 (11.5–22)	17 (12–26.8)	15.2 (11.4–21)	0.21
3-nitrotyrosine, nM	34.5 (20.8–45.6)	84.5 (40.6–127.1)*	125.2 (106.6–151.6)	59.4 (33–105.1)	< 0.0001
K_s_, × 10^−9^cm^2^	6.9 (6.1–7.8)	4.6 (3.7–5.5)*	3.86 (3.22–4.69)	4.84 (4.2–5.88)	< 0.0001
CLT, min	93 (78–99)	108 (98–129)*	116 (100–145)	107 (97–123)	0.048

Abbreviations: *AF*, atrial fibrillation; *MI*, myocardial infarction; *ACEI*, angiotensin-converting enzyme inhibitors; *WBC*, white blood cell count; *eGFR*, estimated glomerular filtration rate; *citH3*, citrullinated histone H3; *NAP-2*, neutrophil-activating peptide 2; *ETP*, endogenous thrombin potential; *TAFI*, thrombin activatable fibrinolysis inhibitor; *PAI-1*, plasminogen activator inhibitor type 1; *K*_*s*_, fibrin clot permeability; *CLT*, clot lysis time

^*^p < 0.05 compared to healthy controls

Median NAP-2 concentration was 89% higher than in healthy controls (*p* < 0.0001). NAP-2 levels were not associated with age, sex, BMI, CHA_2_DS_2_-VASc or HAS-BLED scores. Similarly, types of AF or current sinus rhythm had no influence on NAP-2 levels (data not shown). Patients receiving statins or antiplatelet medications had similar NAP-2 levels compared to individuals without such treatment (all *p* > 0.05). AF patients on angiotensin-converting enzyme inhibitors (ACEI) had slightly lower NAP-2 concentrations compared to those who did not use these agents (607 [424–770] vs. 656 [488–812] ng/ml, *p* = 0.022). Prior stroke, history of MI, diabetes mellitus or hyperlipidemia concomitant to AF were not associated with higher NAP-2 concentrations (all *p* > 0.05).

NAP-2 in the top quartile (> 796 ng/ml) was more often observed in males than females (Table 1). AF patients with NAP-2 levels in the top quartile compared to the remainder were characterized by 13.6% higher white blood cell (WBC) count and 31.7% higher neutrophil count (Table 1). They had also 20.8% higher fibrinogen, but not CRP levels. Importantly, NAP-2 in the top quartile was associated with markedly higher citH3 concentrations (by 86%) and 3-nitrotyrosine levels (by 111%) (both *p* < 0.0001). Of note, 3-nitrotyrosine levels were 49.5% lower in patients in sinus rhythm compared to non-sinus rhythm (50.2 [29.2–112.5] vs. 101.4 [36.5–135.5] nM, *p* = 0.0047).

**Fig. 1:**
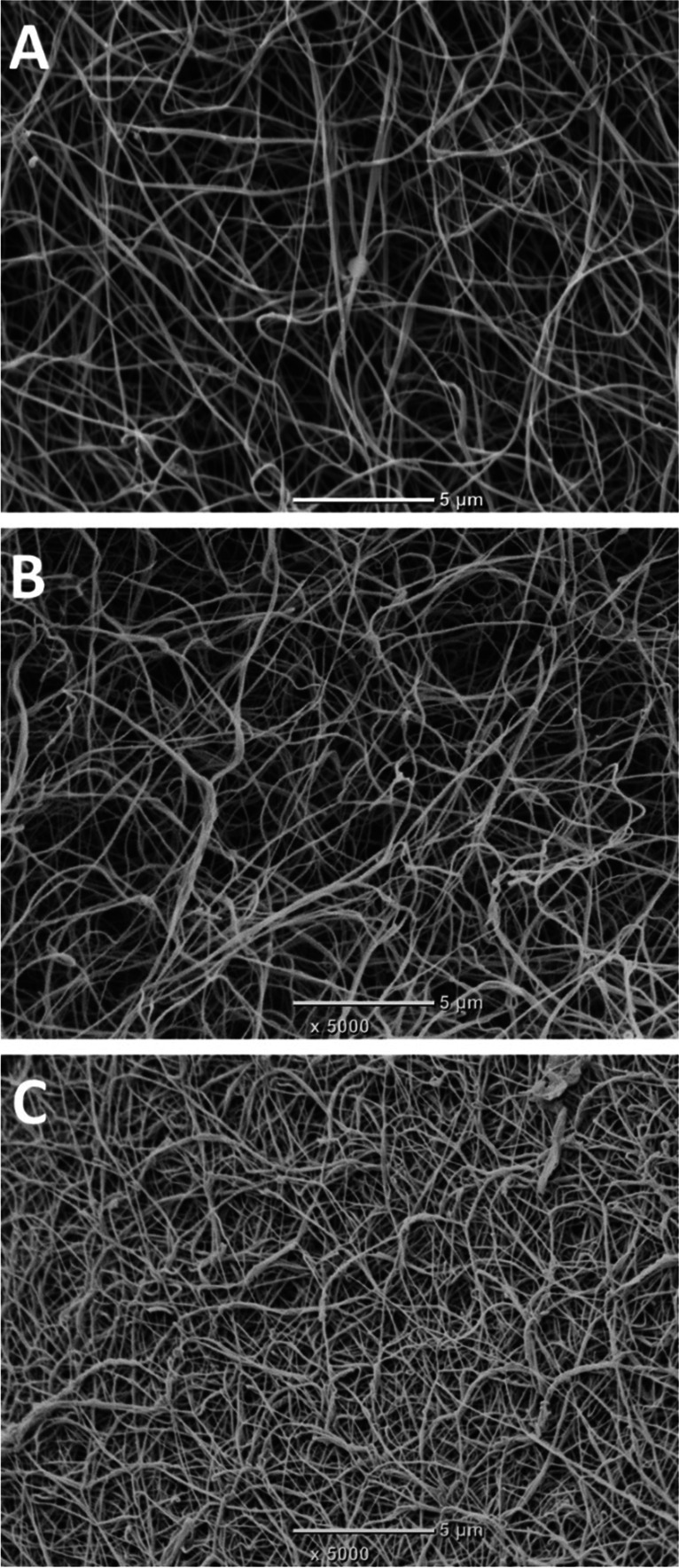
Representative scanning electron microscopy images showing fibrin clot structure in a healthy 65-year-old subject (**A**), a 67-year-old AF patient with NAP-2 level in the bottom quartile (398 ng/ml) (**B**), and a 66-year-old AF patient with NAP-2 level in the top quartile (897 ng/ml) (**C**). Magnification, 5000x. Scale bar, 5 µm

In patients with NAP-2 in the top quartile compared to the remainder, we observed unfavorably altered, prothrombotic fibrin clot properties as reflected by 20.2% lower K_s_ and 8.4% prolonged CLT without any differences in thrombin generation capacity or levels of fibrinolysis inhibitors (Table 1). Along with increasing NAP-2 plasma levels, we observed increased fibrin fibers density, reduced clot porosity and formation of thinner fibrin fibers (Fig. 1).

Thinner fibrin fibers were observed in individuals with NAP-2 in the top quartile compared to those with median NAP-2 levels and to those in the bottom quartile (71 [53–65] vs. 94 [77–101] vs. 102 [96–126] nm, respectively, p < 0.001 for ANOVA).

**Fig. 2:**
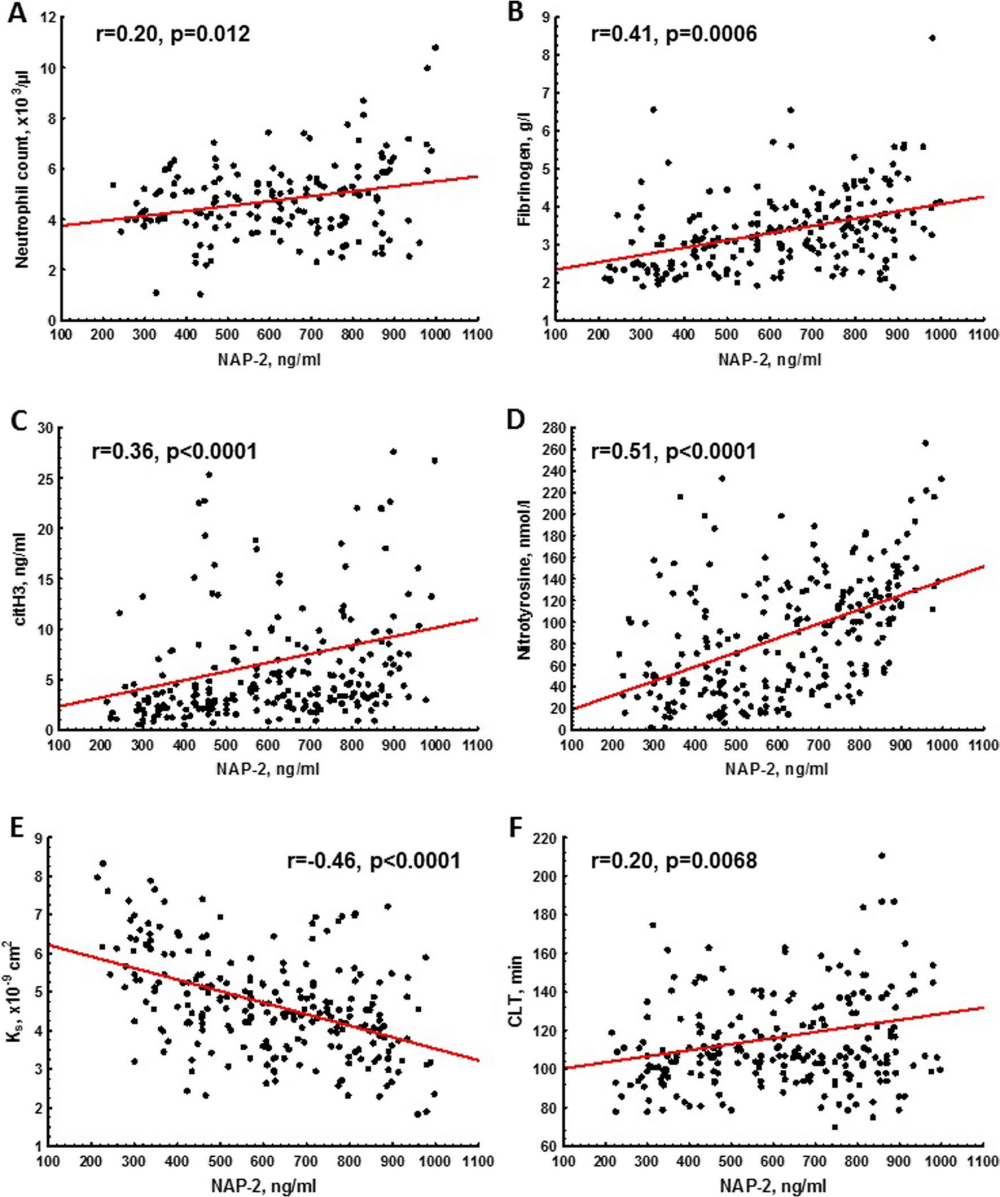
Associations of NAP-2 with neutrophil count (**A**), fibrinogen (**B**), citrullinated histone H3 (citH3; **C**), 3-nitrotyrosine (**D**) levels, fibrin clot permeability (K_s_; **E**), and clot lysis time (CLT; **F**)

NAP-2 levels were positively associated with neutrophil count (r = 0.20, p = 0.012; Fig. 2A) and fibrinogen levels (r = 0.41, p = 0.0006; Fig. 2B), but not with platelet count or P-selectin (both p > 0.05). Of note, positive associations between NAP-2 and fibrinogen concentrations (r = 0.65, p < 0.01), platelet count (r = 0.69, p < 0.01), and P-selectin levels (r = 0.24, p < 0.05) were observed in healthy individuals.

**Fig. 3:**
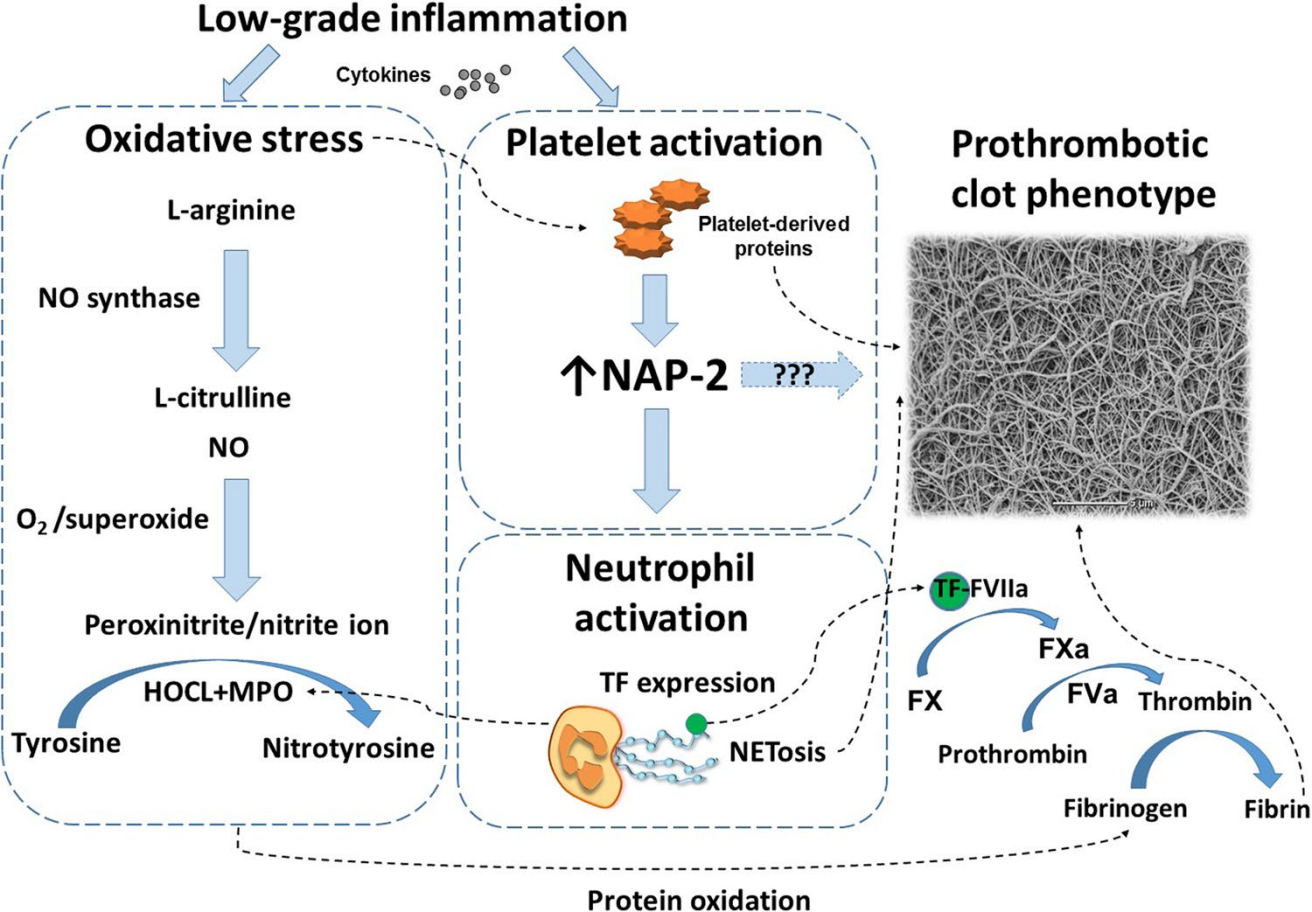
A contribution of NAP-2 to prothrombotic fibrin clot phenotype. MPO, myeloperoxidase; NO, nitric oxide; TF, tissue factor; F, factor

In AF patients citH3 (r = 0.36, p < 0.0001; Fig. 2C) and 3-nitrotyrosine concentrations (r = 0.51, p < 0.0001; Fig. 2D) correlated positively with NAP-2 levels. Moreover, NAP-2 correlated inversely with K_s_ (r = -0.46, p < 0.0001; Fig. 2E) and positively with CLT (r = 0.20, p = 0.0068; Fig. 2F), also after adjustment for fibrinogen and P-selectin (both p < 0.01). Of note, K_s_ correlated inversely (r = -0.45, p < 0.0001), while CLT (r = 0.19, p = 0.0031) positively with 3-nitrotyrosine levels. We found no associations between NAP-2 and AF duration (r = -0.16, p = 0.12) as well as with peak thrombin or ETP, reflecting thrombin generation capacity (both p > 0.05).

The linear regression adjusted for sex, age, and fibrinogen showed that the use of aspirin or clopidogrel were associated with higher K_s_, while higher concentrations of citH3 and NAP-2 were independently associated with reduced K_s_ (Table 2). Solely citH3 and PAI-1 were identified as predictors of longer CLT (Table 2).

**Table 2:** Determinants of fibrin clot properties in AF patients

Factor/variable	Univariable β (95% CI)	Multivariable* β (95% CI)
Plasma fibrin clot permeability (K_s_)		
Aspirin use	0.44 (-0.19; 0.93)	1.88 (0.88; 2.87)
Clopidogrel use	0.60 (0.30; 0.91)	0.62 (0.12; 1.13)
C-reactive protein, per 1 mg/l	-0.068 (-0.033; -0.102)	
citH3, per 1 ng/ml	-0.071 (-0.052; -0.089)	-0.046 (-0.029; -0.064)
NAP-2, per 100 ng/ml	-0.30 (-0.23; -0.37)	-0.21 (-0.14; -0.28)
3-nitrotyrosine, per 10 nM	-0.10 (-0.07; -0.13)	
Clot lysis time (CLT)		
citH3, per 1 ng/ml	0.81 (0.32; 1.29)	0.56 (0.12–1.00)
PAI-1, per 1 ng/ml	1.59 (1.31; 1.86)	1.51 (1.21–1.81)
TAFI, per 10%	2.58 (0.26; 4.90)	
NAP-2, per 100 ng/ml	3.15 (1.27–5.02)	
3-nitrotyrosine, per 10 nM	1.06 (0.36–1.76)	

Abbreviations: CI, confidence interval; for other abbreviations – see Table 1

^*^adjusted for sex, age, and fibrinogen levels

During follow-up we noted myocardial infarction in 2 patients (0.84%) and retinal artery occlusion in one patient (0.42%). Major bleeding was observed in 1 patient (0.42%). NAP-2 increase was associated with higher risk of thromboembolism (per 100 ng/ml; odds ratio, 2.62, 95% confidence interval: 1.05–4.22) and showed no association with major bleeding.

## Discussion

This study is the first to demonstrate that markedly increased NAP-2 concentrations can be observed in AF patients and their increase is associated with oxidative stress, enhanced NETosis, and also prothrombotic fibrin clot phenotype involving compact fibrin network composed of thinner fibers. Thrombosis promotes leukocyte infiltration into tissues, and NAP-2 represents a major mechanism by which leukocytes are guided through thrombi to sites of vascular injury [13]. To our knowledge, the present study provides the first evidence suggesting that in AF patients NAP-2-driven leukocyte migration is likely to be markedly enhanced, suggesting novel inflammatory mechanisms possibly contributing to prothrombotic tendency and thromboembolic risk (Fig. 3). Of note, NAP-2 related effects are independent from the stroke risk assessed using the CHA_2_DS_2_-VASc score, which might help identify an additional subset of AF patients prone to thromboembolic events despite oral anticoagulation. Although clinical relevance of increased NAP-2 concentrations in AF patients remains to be established, the current data suggests that NAP-2 could be a novel factor involved in the AF pathogenesis and associated thromboembolism, which highlight the complexity of interactions among enhanced oxidative stress, inflammation and prothrombotic state in AF and the need for novel therapeutic approaches beyond anticoagulation to prevent stroke or systemic thromboembolism.

In the current study, the novel finding is that AF patients compared to controls, regardless of AF type (paroxysmal/persistent/permanent) or duration are characterized by almost two-fold higher blood levels of NAP-2, which supports the role of leukocytes in the prothrombotic and interrelated proinflammatory states well recognized in this common arrhythmia [13, 14, 25]. A major source of NAP-2 in plasma are activated platelets, however there have been no association of this marker with both platelet count and soluble P-selectin in AF. NAP-2 correlated with neutrophil count along with NETs formation marker. Similar associations were previously reported in acute ischemic stroke patients [15]. We also found positive correlations of NAP-2 with fibrinogen and 3-nitrotyrosine, which highlight the role of oxidative stress and low-grade inflammation in AF patients. Importantly, our observations indicate that NAP-2 is able to modulate pro-coagulant functions and pro-inflammatory responses not only at site of vascular injury [13, 26, 27], but also in AF by itself prior to thromboembolic events. No differences in circulating NAP-2 as well as citH3 between paroxysmal or persistent AF assessed in patients in sinus rhythm and during AF suggest that systemic mechanisms unrelated to the currently observed arrhythmia drive NAP-2-mediated harmful effects. These findings are in line with previously published data, indicating that AF patients despite being in sinus rhythm have impaired coagulation and fibrinolysis [28], supporting currently accepted way of long-term thromboembolic risk assessment indicating the need for anticoagulation in patients with AF which does not take into account type of AF or current heart rhythm.

Similarly, no impact of comorbidities being the established risk factors for AF and thromboembolism [22], supports the concept that NAP-2 reflects multidimensional inflammatory dysregulation in AF. Given the role of neutrophils in the propagation of arterial thrombosis [25], it seems to be of importance whether the measurement of circulating NAP-2 levels could be clinically relevant, especially in follow-up studies. The same holds true for therapies aimed at modulating NAP-2 and suppression of neutrophil-driven thrombosis.

Oxidative stress, reflected by reactive oxygen species (ROS) generation has been considered as a factor triggering both electrical changes in AF and prothrombotic state through activation of the endothelium, platelets and blood coagulation [29]. Increased 8-isoprostane levels, the end-product of non-cyclooxygenase oxidative modifications of arachidonic acid and low density lipoproteins (LDLs), partly through altered fibrin clot structure are associated with thromboembolic events despite anticoagulant therapy in AF patients [5]. Oxidative damage has also been demonstrated in patients with permanent AF, including increased expression of 3-nitrotyrosine within myofibrillar isolates, which may contribute to atrial contractile dysfunction in AF [30]. Ishiyama et al. [31] have demonstrated in a rat model that tyrosine nitration by peroxynitrite resulted in 3-nitrotyrosine formation and myocardial injury. In AF, increased 3-nitrotyrosine levels have been found in blood [32] as well as in cardiomyocytes and endothelial cells of the right atrium compared to controls with no documented AF or AF history [33]. Similarly to previous observations, our study showed that 3-nitrotyrosine levels in patients with maintained sinus rhythm at enrollment are about 50% lower compared to remainders, however, 3-nitrotyrosine levels were still elevated when compared to healthy controls. Thus, in our opinion increased 3-nitrotyrosine levels could result or be implicated in electrical and/or structural remodeling in AF, which highlights the value of current concept of atriopathy.

We are the first to show associations of high plasma NAP-2 with markedly elevated 3-nitrotyrosine levels in AF, which links enhanced leukocyte migration with nitration and its functional consequences. Nitration of fibrinogen also affects tyrosine residues, resulting in 3-nitrotyrosine formation [6]. Bijak et al. [34] have shown that fibrin fibers diameter was thinner and associated with increased clot density after nitration with a high concentration of peroxynitrite. In our study, reduced K_s_ was associated with increased concentrations of 3-nitrotyrosine in AF patients, which provides additional evidence that increased protein nitration is involved in a prothrombotic tendency in AF. In particular, nitration of fibrinogen, which reflects active inflammatory state and oxidative stress, could be of importance in this context [35] by favoring the formation of compact and resistant to lysis fibrin clots. However, the extent of fibrinogen nitration in the presence of elevated NAP-2 in AF patients remains to be explored. We found no associations between NAP-2 levels and peak thrombin or ETP, suggesting that NAP-2 has a minor contribution to thrombin generation capacity. NAP-2-driven neutrophil activation leads to release of NETs, which express TF, contributing to enhanced thrombin generation, especially in acute states. However, due to no association between thrombin generation capacity and citH3 levels the contribution of NAP-2 to thrombin generation seems to be of minor importance in AF.

It is known that the so-called prothrombotic fibrin clot phenotype, as evidenced by formation of compact fibrin clots resistant to lysis, can be observed in patients with AF, regardless of the AF type and CHA_2_DS_2_-VASc score [28, 36, 37] and importantly with the risk of ischemic stroke or systemic thromboembolism despite anticoagulation [9–11]. There have been associations of platelet activation markers, such as platelet factor 4, P-selectin, and soluble CD40L with fibrin clot properties [38, 39]. However, association of elevated NAP-2 with the key marker of fibrin network density, K_s_, in AF patients is novel. Our findings indicate that modulation of fibrin-related prothrombotic state markers in AF by activated platelets and neutrophils is much more complex than thought previously and the significance of NAP-2 in this context deserves investigation.

Regarding potential drug-induced effects, we observed that AF patients treated with ACEIs had lower NAP-2 levels as compared to those without ACEIs treatment. This novel observation is in line with the beneficial effect of ACEIs on the incidence of AF largely by their anti-inflammatory and antioxidant properties [40]. It has been reported that angiotensin II contributes to NETs generation [41], and that ACEIs can improve fibrin clot properties [42] as well as inhibit NETosis. Statins and aspirin appear not to affect NAP-2 concentrations in AF patients, suggesting that most cardiovascular drugs used in AF patients do not alter NAP-2 expression. However, Smith et al. [16] reported in 12 healthy controls that aspirin reduced plasma levels of NAP-2, while 6-month statin therapy increased NAP-2 levels in 35 patients with coronary artery disease. It might be speculated that in AF such effects are abolished, including patients with concomitant cardiovascular disease. Specific therapies targeting NAP-2 may be beneficial for AF patients, but this concept remains to be proven.

This study has several limitations. First, the study size was limited. However, the cohort of AF patients represented typical real-life AF patients, while apparently healthy individuals of similar age, sex, and BMI who did not take any medications were difficult to recruit. Second, fibrin clot properties and all biomarkers were assessed only once at enrolment. We are aware to the fact that blood concentrations of the studied parameters could change over time. Moreover, we did not establish other inflammatory biomarkers known to be associated with cardiac fibrosis or remodeling in AF [43]. It should be noted that associations not necessarily mean the cause-effect relationship, therefore mechanistic studies are required to prove the causative role of NAP-2 in a prothrombotic state in AF, Finally, long-term follow-up is needed to assess the role of elevated NAP-2 concentrations in predicting clinical outcomes in AF patients during anticoagulant therapy.

Taken together, in AF patients markedly elevated NAP-2 levels unrelated to platelet count are associated with unfavorably altered fibrin clot properties, enhanced oxidative stress, and NETosis. Further studies are needed to evaluate whether NAP-2 may represent a novel biomarker associated with inflammatory response and unfavorable clinical outcomes in AF.

## Data Availability

The data that support the findings of this study are available from the corresponding author upon reasonable request.
